# Synthesis and Characterization of Curcumin-Loaded Nanoparticles of Poly(Glycerol Sebacate): A Novel Highly Stable Anticancer System

**DOI:** 10.3390/molecules27206997

**Published:** 2022-10-18

**Authors:** Alessio Massironi, Stefania Marzorati, Alessandra Marinelli, Marta Toccaceli, Stefano Gazzotti, Marco Aldo Ortenzi, Daniela Maggioni, Katia Petroni, Luisella Verotta

**Affiliations:** 1 Department of Environmental Science and Policy, Università degli Studi di Milano, Via Celoria 2, 20133 Milano, Italy; 2 Department of Biosciences, Università degli Studi di Milano, Via Celoria 26, 20133 Milano, Italy; 3 Department of Chemistry, Università degli Studi di Milano, Via Golgi 19, 20133 Milano, Italy

**Keywords:** curcumin, poly(glycerol sebacate), nanoparticles, drug delivery system, human cervical cancer

## Abstract

The research for alternative administration methods for anticancer drugs, towards enhanced effectiveness and selectivity, represents a major challenge for the scientific community. In the last decade, polymeric nanostructured delivery systems represented a promising alternative to conventional drug administration since they ensure secure transport to the selected target, providing active compounds protection against elimination, while minimizing drug toxicity to non-target cells. In the present research, poly(glycerol sebacate), a biocompatible polymer, was synthesized and then nanostructured to allow curcumin encapsulation, a naturally occurring polyphenolic phytochemical isolated from the powdered rhizome of *Curcuma longa* L. Curcumin was selected as an anticancer agent in virtue of its strong chemotherapeutic activity against different cancer types combined with good cytocompatibility within healthy cells. Despite its strong and fascinating biological activity, its possible exploitation as a novel chemotherapeutic has been hampered by its low water solubility, which results in poor absorption and low bioavailability upon oral administration. Hence, its encapsulation within nanoparticles may overcome such issues. Nanoparticles obtained through nanoprecipitation, an easy and scalable technique, were characterized in terms of size and stability over time using dynamic light scattering and transmission electron microscopy, confirming their nanosized dimensions and spherical shape. Finally, biological investigation demonstrated an enhanced cytotoxic effect of curcumin-loaded PGS-NPs on human cervical cancer cells compared to free curcumin.

## 1. Introduction

Cancer is still the second leading cause of death in the world after cardiovascular diseases and, despite the continued efforts of scientists, its incidence and mortality rates have not yet been stopped [1]. Therefore, pursuing novel strategies and less toxic cancer treatments still remains a major challenge for the scientific community. 

Researchers, to improve the selectivity and bioavailability of chemotherapeutic agents, have developed several drug delivery systems (DDSs) designed to allow secure transport to the selected target, thus providing molecules protection against elimination while minimizing drug toxicity to non-target cells [2,3,4,5,6]. During their administration, anticancer agents are generally distributed non-specifically throughout the body, affecting both tumor and healthy cells, resulting in inefficient treatment due to excessive side effects and low internalization of anticancer agents into tumor tissues [7]. On the contrary, among several advantages over traditional chemotherapeutic administration, DDSs can deliver a drug more selectively to a specific target site with less frequent and less concentrated dosing [5]. Moreover, in cancer cells, the passive diffusion is maximized due to the presence of abnormal vascular architecture necessary to serve fast-growing cancer cells. Such phenomenon is named enhanced permeation and retention effect (EPR effect) [6,8]. Accordingly, DDSs may exploit the EPR effect thanks to the nanometer size of the carrier which enhances their penetration and thus incorporation into the tumor target thanks to leaky vasculature [8]. 

**Figure 1:**
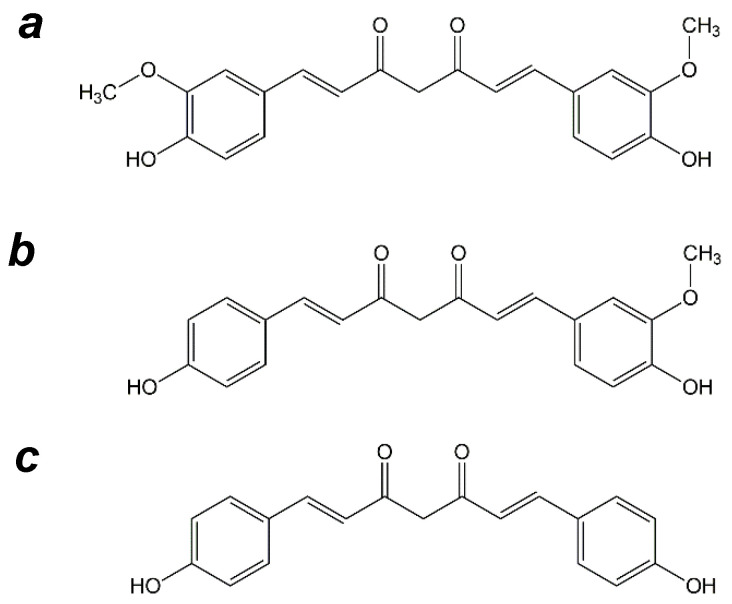
Curcuminoids structures (**a**) curcumin, (**b**) demethoxycurcumin and (**c**) bis-demethoxycurcumin.

Thanks to their unique physicochemical versatility, biodegradability and biocompatibility, biodegradable polymers are widely investigated for the preparation of micro- and nanoparticles as drug carriers for anticancer agents [3,9]. Among the polymers that can be used for this purpose, poly(glycerol sebacate) (PGS) has received notable attention due to its unique physical features [10]. Both monomers used for PGS synthesis via polycondensation, i.e., glycerol and sebacic acid, are bio-based, biocompatible and approved by the Food and Drug Administration. PGS synthesis is inexpensive, and the obtained product is generally soft and has flexible mechanical properties that make it suitable for working with soft tissue and organs in a mechanically dynamic environment [11]. Originally designed as a biodegradable polymer with improved elastic mechanical properties and biocompatibility, research on PGS-based medical applications has uncovered several unique properties that have bolstered its use as a biomaterial [12]. Nowadays, PGS is commonly exploited to develop 3D structures such as scaffolds for tissue engineering [13] but its possible application as a nanodelivery system has been explored less [14,15]. Moreover, several studies demonstrate that the hydrophobicity degree of a carrier is one of the major determinants to achieve correct and complete drug delivery in specific environments (e.g., lymphatic system) [4,14,15,16]. In this context, the aim of the present study was the development of highly stable PGS nanostructures designed to ensure secure loading of curcumin extract (standardized as 95% in curcuminoids composed by: curcumin 85%, demethoxycurcumin 14% and bis-demethoxycurcumin 1%) (Figure 1) as a novel anticancer system. Curcumin, [1,7-bis(4-hydroxy-3-methoxyphenyl)-1,6-heptadiene-3,5-dione], a naturally occurring polyphenolic phytochemical isolated from the powdered rhizome of *Curcuma longa*, was selected as an anticancer agent in virtue of its strong chemotherapeutic activity against different cancers types combined with good cytocompatibility against healthy cells [17,18,19]. 

Several studies indicate that curcumin can modulate all kinds of cancer hallmarks, including uncontrolled cell proliferation, cancer-associated inflammation, cancer cell death, signaling pathways, cancer angiogenesis, and metastasis [20]. However, its possible exploitation as a novel chemotherapeutic is hampered by its low water solubility [21,22,23], which results in poor absorption and low bioavailability upon oral administration; its possible encapsulation within PGS nanoparticles represents a novel and promising administration alternative combining its strong anticancer activity with the biocompatibility and high hydrophobicity of the polymer. Nanoprecipitation was selected as the formulation technique since it offers several advantages for producing smaller nanoparticles with narrow unimodal size distribution [24]. Furthermore, it is scalable and rapid to perform [25]. The obtained NPs were characterized through dynamic light scattering and transmission electron microscopy analysis to evaluate nanoparticle size and morphology and monitoring their stability over time. 

Cervical cancer is the leading cause of death for cancer in women resulting in over 340,000 deaths worldwide [26]. Most cases of cervical cancer can be attributed to persistent human papilloma virus (HPV) infection, which is preventable thanks to safe and effective anti-HPV vaccination. Screening programs allow the identification of cervical pre-cancer lesions, thus allowing prompt treatment and cure of early stage cervical cancer with surgical interventions, chemotherapy and/or radiotherapy [27]. However, recurrent cervical cancer due to resistance to chemotherapy still represents a major challenge. In this context, polyphenols, such as nanoencapsulated curcumin, are currently viewed as a possible adjuvant therapy for overcoming chemoresistance in cancer cells, since they affect multiple targets, including cell death [28]. Therefore, the biological activity of the PGS nanosystem has been investigated in vitro against human cervical cancer HeLa cells to evaluate the pro-apoptotic anticancer activities of curcumin-loaded PGS-NPs compared to free curcumin. Our results show that curcumin-loaded PGS-NPs display higher cytotoxicity, anti-HPV activity and can activate apoptosis in HeLa cervical cancer cells.

## 2. Results

### 2.1. Synthesis and Characterization of Poly(Glycerol Sebacate)

**Scheme 1:**
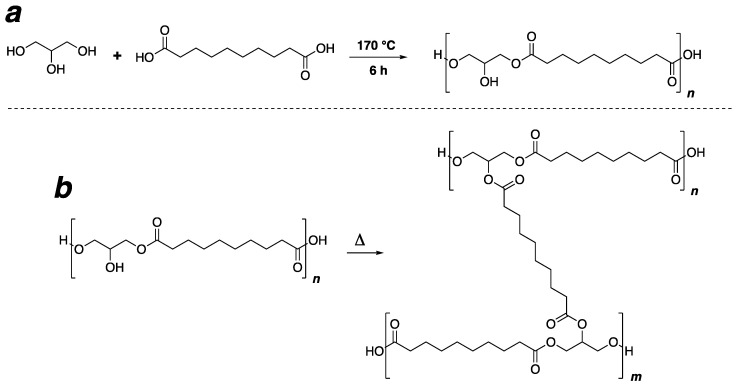
Synthesis of PGS. (**a**) synthesis of linear PGS through polycondensation of glycerol and sebacic acid. (**b**) curing reaction to yield a thermoset.

PGS was synthesized via a polycondensation reaction between glycerol and sebacic acid to first form a linear aliphatic polyester (Scheme 1a) that can be further cured to yield a crosslinked thermoset elastomer (Scheme 1b).

The main hurdle in the synthesis of PGS derives from the nature of the monomers. While glycerol can be described as an A3 type monomer, sebacic acid is a difunctional species that can be described as a B2 type monomer. This difference in the number of reactive functionalities between the two molecules results in difficulties in proper control of the reactivity during the polycondensation reaction. In this regard, even if it is true that the primary alcohol groups in glycerin are more reactive than the secondary ones, a non-proper management of the reaction conditions can result in the formation of an undesired crosslinked material. Literature reports on the synthesis of PGS usually rely on an equimolar mixture of the two monomers, to target a linear polyester. The reaction is carried out for very long times at low temperatures, in order to limit the occurrence of crosslinking [29]. During this step, the molecular weight of the linear segments has limited control through different reaction times. After this first condensation step, the crosslinking reaction is carried out to yield a thermoset. The mechanical properties of the crosslinked product can be controlled through the curing conditions that can give access to a significant amount of different products with different properties [30].

This classical procedure for the synthesis of PGS requires long times, lasting several days. On the contrary, the procedure reported in this paper is based on a different approach. The two monomers are loaded in different molar amounts, with an excess of sebacic acid to partially make up for the excess of alcoholic functionalities. The reaction was carried out for 6 h at 170 °C resulting in a linear polymer in the form of a waxy solid that was further employed for the preparation of nanoparticles.

PGS was characterized through size exclusion chromatography (SEC) for the determination of the molecular weight and polydispersity. Molecular weight data of the polymer were detected as follows: (M_n_) = 3100 Da; (M_w_) = 12,000 Da; D = 4.0, expressed as polystyrene equivalents. The high polydispersity of the polymer was attributed to the occurrence of branching reactions. These kinds of reactions are competitive with a linear chain growth and affect the microstructure of the product resulting in a complex mixture of species. However, the product was filtered before the analysis in order to eliminate every possible crosslinked fraction.

DSC analysis pointed out the semicrystalline nature of the polymer, with T_g_ = −11.3 °C; T_m_ = 8.1 °C.

NMR and FT-IR were also carried out in order to depict the structural features of the polymer. Spectral data were in good agreement with the literature [31]. Spectra are shown in the supporting information.

### 2.2. PGS-NPs Fabrication

**Figure 2:**
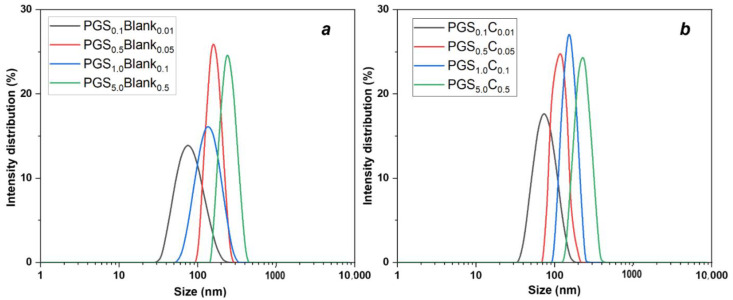
DLS size distribution (intensity) of unloaded (**a**) and curcumin-loaded PGS-NPs (**b**) formed at different polymer concentrations (0.1, 0.5, 1.0 and 5.0 mg/mL).

Loaded and unloaded PGS-NPs were prepared by nanoprecipitation according to a general procedure [24], where an acetone solution of PGS and curcumin was precipitated in deionized water allowing the formation of NPs. In order to evaluate the effect of polymer concentration over nanoparticles stability and size, PGS-NPs were formulated at different polymer concentrations in the starting organic solution (before nanoprecipitation); namely, PGS_0.1_C_0.01_, [PGS]: 0.1 mg/mL; PGS_0.5_C_0.05_, [PGS]: 0.5 mg/mL; PGS_1.0_C_0.1_, [PGS]: 1.0 mg/mL; PGS_5.0_C_0.5_, [PGS]: 5.0 mg/mL by keeping constant the curcumin-to-PGS ratio (curcumin/PGS ratio equal to 10% by weight). Concentration values refer to the final suspension water volume, after nanoprecipitation and after ethanol evaporation. For unloaded PGS-NPs (reported as Blank in Table 1) the same procedures, as well as the same PGS concentrations, were used but in the absence of curcumin. All the adopted experimental conditions led to the formation of monodisperse nanosized (loaded and unloaded) PGS particles, displaying a narrow monomodal size distribution in the nanometer range, as shown in Figure 2. Table 1 reports the maxima values of the distribution curves together with the polydispersion index (PdI) derived from the cumulant analysis.

Results relevant to individual experiments are reported in Table 1. It was observed that the composition of the organic phase, determined by PGS and curcumin concentration, strongly affected the diameter distribution of the particles. Increasing the polymer concentration increased the PGS-NPs diameter.

**Figure 3:**
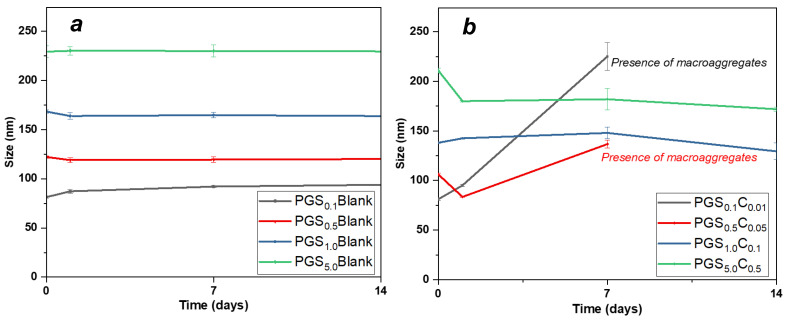
Hydrodynamic diameter of blank (**a**) and curcumin-loaded (**b**) PGS-NPs over time from sample preparation.

On the contrary, the presence of curcuminoids seemed to not influence the formation of the NPs since only slight differences in terms of NP size were observed as a consequence of their loading. However, curcumin strongly affected particles stability, since all unloaded PGS-NPs (reported as Blank in Table 1) demonstrated good stability up to 14 days from the preparation of the samples, while an increase in size and polydispersity of the two curcumin-loaded samples (namely PGS_0.1_C_0.01_ and PGS_0.5_C_0.05_) was observed after 7 days from samples preparation (Table 1 and Figure 3). After 7 days, both formulations presented macroscopic yellow aggregates indicating the loss of their colloidal stability.

On the contrary, curcumin-loaded samples (PGS_5.0_C_0.5_ and PGS_1.0_C_0.1_) demonstrated better stability over 14 days, where only slight reductions in particles size distribution were observed, thus comparable to the blank formulations.

The encapsulation efficiency of the most promising formulations (PGS_1.0_C_0.1_ and PGS_5.0_C_0.5_ purified by centrifugation) were equal to 99.8% (as measured by ultra-high-performance liquid chromatography, UPLC) for both developed nanoparticles, confirming the complete encapsulation of the active compound within the polymeric matrix. These values were particularly high compared to those reported in the literature, where the encapsulation efficiency of curcumin-loaded NPs spanned from 5% to 95% [32,33,34]. Since almost the whole curcumin was entrapped into the NPs, no further purification of colloidal suspensions was deemed necessary. Most importantly, no additives such as Tween-80 or PEG, commonly used to maximize the loading, were employed [35,36]. 

Due to the high proven stability, two formulations (namely PGS_1.0_C_0.1_, PGS_5.0_C_0.5_) as well as the corresponding unloaded formulations (PGS_1.0_Blank and PGS_5.0_Blank) were selected as the most promising candidates for the in vitro release studies and biological assays. 

### 2.3. PGS-NPs Stability Analysis in Cells Culture Medium

**Figure 4:**
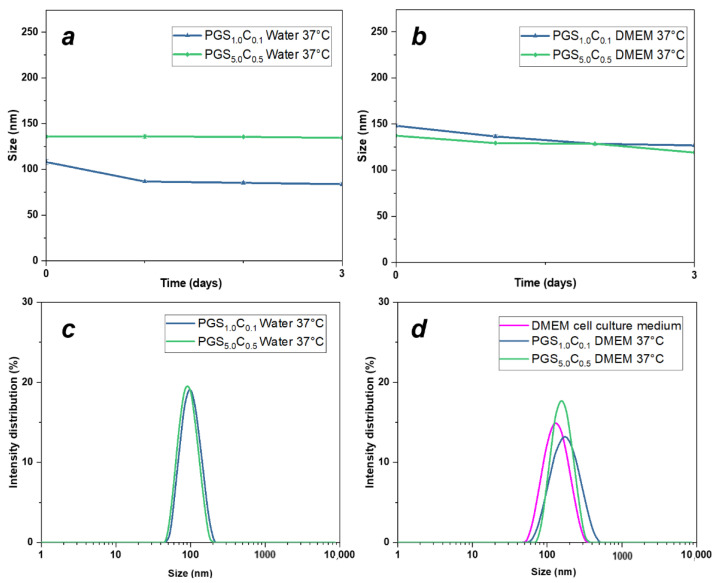
Hydrodynamic diameter of curcumin-loaded PGS-NPs over time from sample preparation in water (**a**) and in DMEM (**b**). DLS size distribution (intensity) of selected loaded PGS-NPs after their dilution in deionized water (**c**) and DMEM cell culture medium (**d**) at 37 °C.

The colloidal stability of PGS_1.0_C_0.1_ and PGS_5.0_C_0.5_ was investigated in cells culture medium by diluting the samples in water at 37 °C and in Dulbecco’s Modified Eagle Medium (DMEM) supplemented with fetal bovine serum (FBS) at the highest concentration tested during the planned biological investigation ([PGS]:0.2 mg/mL). A reduction in particle diameter was observed for both nanosystems (Table 2), possibly due to the more diluted conditions. Indeed, the higher dilution can cause NPs to be on average more distant from one another, giving rise to a less probable interaction among particles over time in comparison to higher concentrations where collisions and interactions are maximized. After a 24 h incubation in water at 37 °C, a reduction in particle size distribution was observed for PGS_1.0_C_0.10_, suggesting lower stability at higher temperatures compared to PGS_5.0_C_0.5_, where no differences were observed even at 37 °C after 7 days of incubation. The results of particle stability tests in cell culture medium and deionized water at 37 °C are reported in Table 2 and the monitoring of size over time is shown in Figure 4.

The same experiment was conducted in cell culture medium. The background noise of cell culture medium caused an increase in the polydispersity index for all the analyzed samples. Indeed, a broad particle distribution of circa 120 nm was observed by DLS analysis of the sole DMEM. This may be due to the presence of several species in the medium such as amino acids, antibiotics and in particular of fetal bovine serum (FBS) which could strongly affect the particle size analysis. The DLS analysis of PGS_1.0_C_0.1_ NPs was heavily affected by the background noise of DMEM and did not allow for correct particle size distribution measurements. On the contrary, PGS_5.0_C_0.5_ revealed the presence of homogeneous particle size distribution with an unchanged maximum compared to the one observed in pure water, thus confirming the good colloidal stability even in the cell medium. In this context, PGS_5.0_C_0.5_ was selected as the most promising curcumin-loaded preparation to test anticancer activity. Our results evidence the importance of particle size since bigger PGS-NPs ensured better stability of the whole system, especially when encapsulating curcumin.

### 2.4. PGS-NPs Morphological Characterization

**Figure 5:**
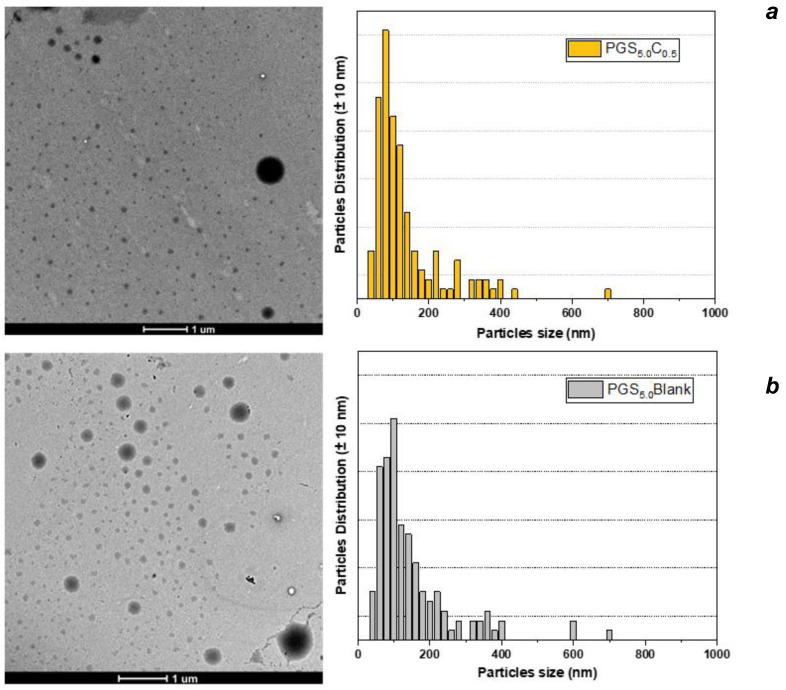
PGS_5.0_C_0.5_ (**a**) and PGS_5.0_Blank (**b**) TEM images and relevant size distribution graphs calculated from TEM images.

The selected PGS_5.0_C_0.5_ and PGS_5.0_Blank samples were characterized by transmission electron microscopy, confirming their spherical shape (Figure 5). The dimensions of PGS_5.0_C_0.5_ and PGS_5.0_Blank estimated by TEM analysis indicated an average size of 121 ± 11 nm and 124 ± 13 nm, respectively. Moreover, TEM analysis allowed confirmation of monomodal size distribution for both developed NPs. A few large aggregates were present in the micrographs as black spots of higher size compared to most of the obtained PGS-NPs, possibly formed during the particle deposition on formvar grids.

### 2.5. Curcumin Release in Cell Culture Medium

**Figure 6:**
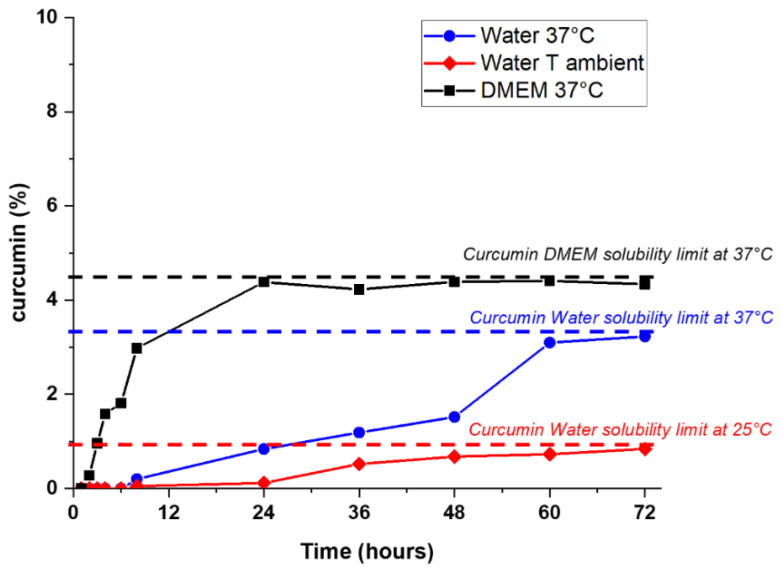
Release kinetics of curcumin from PGS_5.0_C_0.5_ NPs in DMEM at 37 °C (blank), water at 37 °C (blue), and water at room temperature (red).

The experimental solubility of curcumin in water at room temperature, at 37 °C and in DMEM at 37 °C were determined by UPLC as 2 × 10^−4^ mg/mL, 7 × 10^−4^ mg/mL and 1 × 10^−3^ mg/mL, respectively. Then, curcumin release from developed nanostructures was investigated. Figure 6 shows the release profile of curcumin from PGS_5.0_C_0.5_ nanoparticles through the dialysis membrane and curcumin solubility limit in medium. After 24 h, 4.4% of the total loaded curcumin was released from PGS_5.0_C_0.5_ in DMEM at 37 °C, reaching the experimental curcumin solubility limit in the medium (black dotted line in Figure 6). On the other hand, due to the lower solubility of curcumin in water, the plateaus corresponding to 3.2% and 0.8% of release were reached after 60 h in water at 37 °C and room temperature, respectively. The low release of curcumin from PGS_5.0_C_0.5_ was ascribable to the low solubility of curcumin in aqueous solvents.

### 2.6. Cytotoxic Effects of Unloaded and Curcumin-Loaded PGS-NPs in HeLa Cervical Cancer Cells

**Figure 7:**
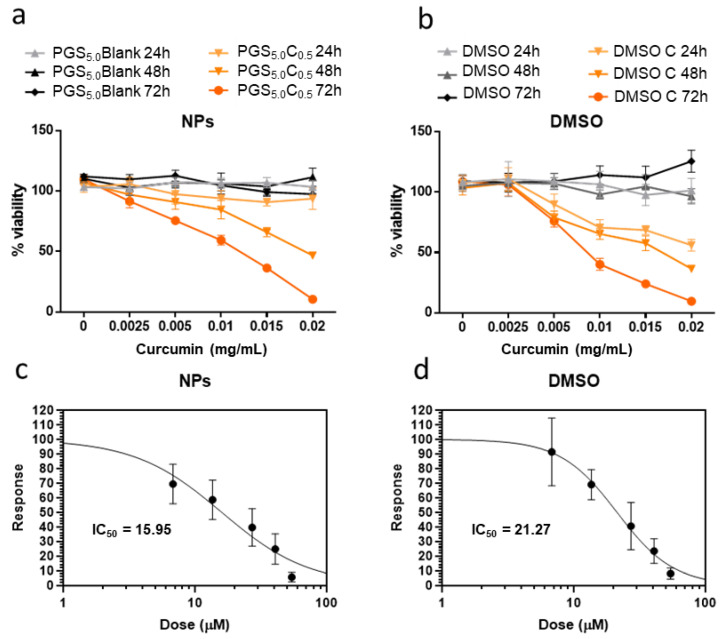
Cytotoxicity assay in HeLa cervical cancer cells with different concentrations of PGS_5.0_Blank and PGS_5.0_C_0.5_ dissolved in sterile dH_2_O (**a**) and of free curcumin dissolved in DMSO (DMSO-C, **b**). Values are means ± SEM (n = 12). IC_50_ values calculated are based on cell viability of HeLa cells at 72 h after treatment with PGS_5.0_C_0.5_ (**c**) and free curcumin dissolved in DMSO (**d**).

HeLa cells were treated with different concentrations of free curcumin dissolved in DMSO and of unloaded (PGS_5.0_Blank) and curcumin-loaded PGS-NPs (PGS_5.0_C_0.5_) for 24, 48 and 72 h (Figure 7). No significant decrease in cell viability was observed when HeLa cells were treated with PGS_5.0_Blank, indicating that PGS-NPs are not cytotoxic even at higher concentrations (Figure 7a). Instead, PGS_5.0_C_0.5_ showed dose-dependent cytotoxicity similar to that obtained with free curcumin dissolved in DMSO (Figure 7b), thus suggesting that the water solubility of curcumin-loaded NPs is similar to that of free curcumin. To better compare the cytotoxic efficacy of curcumin-loaded PG-NPs (PGS_5.0_C_0.5_) vs. free curcumin, the 50% inhibitory concentrations (IC_50_) were calculated. Interestingly, the IC_50_ value of curcumin-loaded PGS-NPs at 72 h (15.95 µM) was significantly lower than that of free curcumin (21.27 µM, Figure 7c,d), suggesting a higher cytotoxic effect of curcumin-loaded PGS-NPs compared to free curcumin. No significant reduction in cell viability was observed when NIH-3T3 healthy fibroblast cells were treated with PGS_5.0_Blank and different concentrations of PGS_5.0_C_0.5_, suggesting that unloaded and curcumin-loaded PGS-NPs have no cytotoxic effect on non-cancerous cells (Figure S5).

### 2.7. Effect of Curcumin-Loaded PGS-NPs in Inducing Apoptosis of HeLa Cells

**Figure 8:**
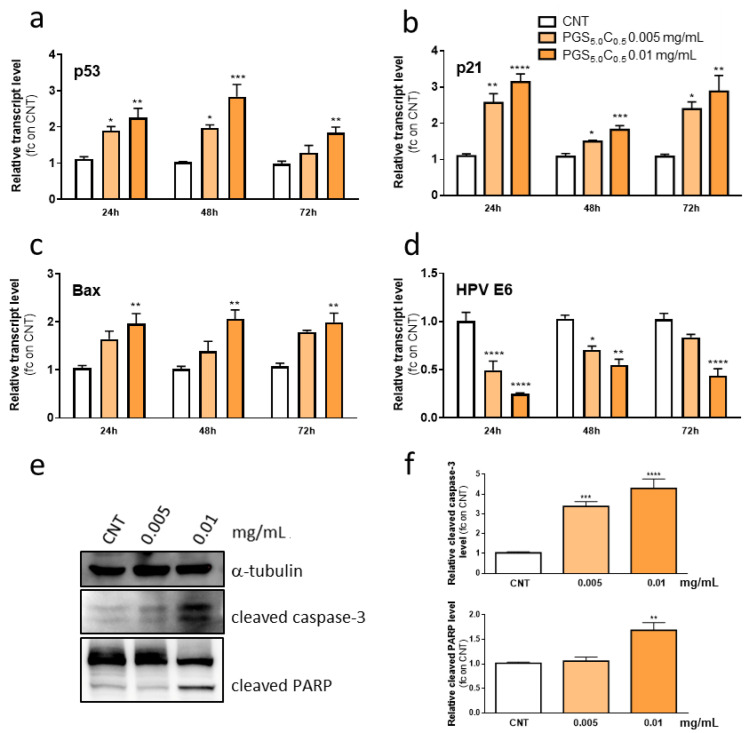
Effect of curcumin-loaded PGS-NPs on the induction of apoptosis. Real-time RT-PCR analysis of transcript levels of p53 (**a**), p21 (**b**), Bax (**c**) and HPV E6 genes (**d**) in HeLa cells treated with 0.005 or 0.01 mg/mL of PGS_5.0_C_0.5_ for 24, 48 and 72 h. Each transcript level is expressed as fold change (fc) with respect to control samples treated with vehicle (CNT). Values are means ± SEM (n = 6) (**e**) Western blot panel and (**f**) relative quantification of cleaved caspase-3 and PARP levels in HeLa cells treated with 0.005 or 0.01 mg/mL of PGS_5.0_C_0.5_ for 72 h. Data were analyzed with ImageJ and normalized to α-tubulin and expressed as fold change over CNT. Values are means ± SEM (n = 3). * *p* < 0.05, ** *p* < 0.01, *** *p* < 0.001, **** *p* < 0.0001 indicate significant differences (two-way ANOVA followed by Tukey’s multiple comparisons test) versus CNT. CNT (blank): Control; PGS_5.0_C_0.5_ (light orange) [PGS] 0.005 mg/mL [Curcumin] 0.0005 mg/mL; PGS_5.0_C_0.5_ (dark orange) [PGS] 0.01 mg/mL [Curcumin] 0.001 mg/mL).

The induction of apoptosis in HeLa cells treated with 0.005 and 0.01 mg/mL of PGS_5.0_C_0.5_ for 24, 48 and 72 h was then investigated. PGS_5.0_C_0.5_ provoked a dose-dependent up-regulation of genes involved in apoptosis (p53 and Bax, Figure 8a,b) and cell cycle arrest (p21, Figure 8c) compared to non-treated cells. No up-regulation was observed when HeLa cells were treated with PGS_5.0_Blank (data not shown). Western blot analysis confirmed that PGS_5.0_C_0.5_ increased cleaved caspase-3 and PARP levels and induced apoptosis (Figure 8e). A significant reduction of the transcript levels of the viral HPV E6 oncogene was also observed, suggesting an inhibitory effect of PGS_5.0_C_0.5_ on its expression (Figure 8d). Overall, these findings suggest that PGS_5.0_C_0.5_ NPs are capable of inducing apoptosis by activating cell cycle arrest and apoptosis in HeLa cervical cancer cells.

## 3. Materials and Methods

### 3.1. Materials

Glycerol and sebacic acid were purchased from Sigma-Aldrich Chemicals (Milan, Italy). Acetone was purchased from Carlo Erba (Milan, Italy). Curcumin extract (standardized as 95% in curcuminoids composed of: curcumin 85%, demethoxycurcumin 14% and bis-demethoxycurcumin 1%, (chromatograms relevant to curcuminoids UPLC analyses are reported in the supplementary information) was kindly donated by Indena S.p.A. DMEM cell culture medium supplemented with 10% (*v*/*v*) fetal bovine serum was purchased from Sigma-Aldrich, (St. Louis, MO, USA), penicillin and streptomycin from Gibco/BRL (Carlsbad, CA, USA) and L-glutamine from Life Technologies (Carlsbad, CA, USA). HPLC grade acetonitrile, water, and formic acid were purchased from Sigma-Aldrich (Milan, Italy).

### 3.2. Polymer Preparation

#### 3.2.1. PGS Synthesis

Glycerol (0.116 mol, 10.69 g) and sebacic acid (0.145 mol, 29.36 g) were placed in a 250 mL three-necked round bottom flask and placed under nitrogen flow. The reaction was carried out in a closed oven at 170 °C for 6 h, providing mechanical stirring (40 rpm) and then the polymer was left cooling overnight under nitrogen flow.

#### 3.2.2. PGS Characterization

The molecular weight of synthesized polymers was evaluated using a size exclusion chromatography (SEC) system having a Waters 1515 isocratic high-performance liquid chromatography (HPLC) pump and a four Waters Styragel column set (HR3-HR4-HR5-HR2) with a UV detector Waters 2487 Dual λ Absorbance Detector set at 230 nm, using a flow rate of 1 mL/min and 60 μL as the injection volume. The samples were prepared by dissolving 50 mg of polymer in 1 mL of anhydrous CH_2_Cl_2_ and filtering the solution through 0.45 μm filters. Given the relatively high loading, a check was performed using a lower concentration of polymer (5 mg/mL) to verify that no column overloading had occurred. Higher loadings were preferred as the UV signal of PGS was relatively weak. Molecular weight data are expressed in polystyrene (PS) equivalents. The calibration was built using 16 monodispersed PS standards, having a peak molecular weight ranging from 1,600,000 Da to 106 g/mol (i.e., ethylbenzene). For all analyses, 1,2-dichlorobenzene was used as an internal reference.

Molecular weight data of the synthesized PGS were detected as follows: (Mn)^−^ = 3100 Da; (Mw)^−^ = 12,000 Da; D = 3.98. All data are expressed as polystyrene equivalents.

Differential scanning calorimetry (DSC) analyses were conducted using a Mettler Toledo DSC1 on samples weighing from 5 to 10 mg each. Melting and crystallization temperatures were measured using the following temperature cycles: (1) heating from −50 °C to 150 °C at 10 °C/min; (2) cooling from 150 to −50 °C at 10 °C/min; (3) heating from −50 to 150 °C at 10 °C/min. The first two cycles were run to erase the thermal history of the samples. Glass transition temperature (Tg) and melting temperature (Tm) were determined during the second heating scan.

Thermal transition data of the synthesized PGS were detected as follows: Tg = −11.3 °C; Tm = 8.1 °C.

Fourier transform infrared spectroscopy (FT-IR) Using a FT-IR Spectrometer (Spectrum 100, PerkinElmer) with an attenuated total reflection (ATR) was used to register spectra for PGS samples. FT-IR spectrum of PGS is reported in the supporting information file.

^1^H and ^13^C NMR spectra for PGS sample were recorded using a Bruker Ultrashield 400 MHz. The chemical shifts are reported in ppm and referred to TMS as internal standards. All samples were prepared by dissolving 6–8 mg of polymer into 1 mL of DMSO d_6_. Spectra are shown in the supporting information file.

### 3.3. PGS-NPs Preparation

#### 3.3.1. Formulation Method

Curcumin-loaded PGS-NPs were prepared by nanoprecipitation according to a general procedure [24]. Briefly, in a glass vial equipped with a magnetic stirrer, selected amounts of PGS and curcumin powder (curcumin/PGS ratio equal to 10% by weight) were dissolved in 4 mL ethanol (organic phase, Table 1). The resulting mixture was stirred at room temperature until complete dissolution was reached. The organic phase was dropped by means of a 22G needle syringe, kept in vertical position and without piston, in 10 mL of deionized water under moderate stirring. The as-formed NPs were kept under nitrogen flow for 3 h to achieve complete ethanol evaporation, assessed by a 4 mL volume decrease. For unloaded PGS-NPs (reported as Blank in Table 3) the same procedure was repeated in the absence of curcumin.

#### 3.3.2. Procedure Optimization

In order to evaluate the effect of the different formulation parameters on the drug entrapment and release kinetics of curcumin, various formulation parameters were investigated (polymer concentration and purification method). Data relevant to individual experiments are summarized in Table 3. 

### 3.4. Curcumin Encapsulation Efficiency

Encapsulation efficiency (the percentage of drug successfully entrapped into the nanoparticle) was estimated by using Equation (1):

(1) $$Encapsulation\;efficiency=\frac{mg\;of\;encapsulated\;Curcumin}{mg\;of\;total\;Curcumin}\times 100$$

To determine the encapsulation efficiency of PG-NPs, suspensions were firstly ultracentrifuged (VWR Microstar 17 centrifuge, Germany) at 16,000 rpm and 24 °C for 60 min from the aqueous medium containing a known amount of free curcumin. The supernatant was separated, filtered (0.2 µm nylon syringe filters) and injected in a UPLC (ultra-high-performance liquid chromatography) system for released curcumin quantification (chromatographic method described below).

### 3.5. PGS-NPs Morphological Characterization

Most promising unloaded and loaded PGS-NPs were characterized through transmission electron microscopy (TEM). Samples for TEM imaging were prepared by dropping PGS-NPs suspensions on single-side-polished copper grids coated with a formvar film and dried at room temperature. Samples were observed using a Talos L120C transmission electron microscope (Thermo Fisher Scientific, Milan, Italy) equipped with a 4K digital camera Ceta CMOS (Thermo Fisher Scientific). Obtained images were elaborated using Nanoscope software and the particle size distribution was calculated. A minimum of 150 nanoparticles were selected in each acquired TEM image.

Dynamic light scattering (DLS) analysis was carried out by using a Nanozetasizer (Malvern Instruments) equipped with a 4.0 mW He–Ne laser operating at 633 nm and an avalanche photodiode detector in order to determine the average size of the obtained PGS-NPs. All the measurements were repeated at least 3 times. Colloidal stability of PGS-NPs at different concentrations (0.2 and 0.1 mg/mL) was investigated by means of DLS at 37 °C. PGS-NP size was monitored at different times from sample preparation (time 0, 24, 48, 72 h). The same experiment was carried out using the Dulbecco’s Modified Eagle Medium (DMEM, Euroclone, Milano, Italy) supplemented as described below and stored at 37 °C in a humidified 5% CO_2_ atmosphere. 

### 3.6. Curcumin In Vitro Release Studies

Release kinetic studies were performed on PGS_5.0_C_0.5_ nanoparticles by using a dialysis tubing cellulose membrane (molecular weight cut-off = 14,000 Da). Curcumin-loaded NPs were diluted in an appropriate volume of water or DMEM cell culture medium and then placed into dialysis tubes. 5 mL of sample was dialyzed against 20 mL of water or DMEM at 37 °C. At specific time intervals (1, 2, 3, 6, 24, 36, 48, 60 and 72 h), 1 mL of the medium was analyzed by means of UPLC (chromatographic method described below) to determine the amount of released curcumin. Consequently, the same volume of fresh medium was replaced. All the measurements were performed in triplicate. The same experiment was performed at 4 °C and at room temperature to determine the influence of temperature during the curcumin release. The in vitro release profile was measured by using Equation (2):

(2) $$Release_{curcumin}\;\left(\%\right)=\frac{C_{Released\;\left(t\right)}}{C_{Total}}\times 100$$

where *C_released_* represents the concentration (mg/mL) of released curcumin at time point (t), and *C_Total_* (mg/mL) corresponds to the total amount of curcumin loaded into PGS-NPs.

### 3.7. UPLC Analysis

Curcumin was quantified through a Waters ACQUITY UPLC system equipped with a quaternary solvent manager system, autosampler, thermostated column compartment and a PDA detector. The analytical separation was performed using an ACQUITY UPLC^®^ (Waters corp., Milford, MA, USA) BEH C18 column (1.7 μm × 2.1 mm × 50 mm). Curcumin release was analyzed using a mobile phase composed of water (+0.1% of formic acid) (A) and acetonitrile (+0.1% of formic acid) (B). The flow rate was set at 0.25 mL min^−1^ and the linear gradient elution was: 0 min, 70% A; 10 min, 30% A; 11 min, 70% A; with a re-equilibration time of 3 min before the next injection. The column temperature was maintained at 34 °C and the wavelength set at 425 nm, corresponding to the maximum absorption of curcumin. Before samples injections, five dilutions of a curcumin methanolic solution (0.2 mg/mL) were prepared in the range 0.001–0.2 mg/mL. Standard solutions were filtered (0.2 μm nylon filters) and injected three times into the UPLC system. Curcumin was eluted as a sharp peak at retention time of 5.5 min. In the operative concentration range the trend was linear for each compound, with no saturation effects that could bend the linearity. The area under each peak was quantified by instrumental software and plotted versus the concentration. The best fit of experimental data in the plot “Peak area vs. [curcumin]” was then used for released curcumin quantification in each sample. Chromatograms relevant to curcuminoids UPLC analysis are reported in the supplementary information.

### 3.8. Cell Culture Assays—Cytotoxicity Study

#### 3.8.1. Cell Culture and MTT Assay

Human cervical cancer HPV18^+^ HeLa cell lines and the healthy fibroblast NIH-3T3 cell line were cultured at 37 °C in a humidified atmosphere containing 5% CO_2_ in DMEM supplemented with 10% (*v*/*v*) fetal bovine serum (Sigma-Aldrich, St. Louis, MO, USA), 100 Units/mL penicillin, 100 µg/mL streptomycin (Gibco/BRL, Carlsbad, CA, USA) and 2 mM L-glutamine (Life Technologies, Carlsbad, CA, USA). The inhibitory effect of unloaded and curcumin-loaded PGS-NPs on cervical cancer HeLa cells and NIH-3T3 fibroblasts was analyzed by cell viability assay using 3-(4,5-dimethylthiazol-2-yl)-2,5-diphenyltetrazolium bromide (MTT) test as previously described [37]. Briefly, HeLa cells were seeded in a 96-well plate (about 25,000 cells per well for HeLa and 5000 cells per well for NIH-3T3), incubated for attachment for 24 h at 37 °C and then treated with free curcumin dissolved in DMSO and with unloaded and curcumin-loaded PGS-NPs in 12 replicates at different concentrations (0, 0.0025, 0.005, 0.01, 0.015, 0.02 mg/mL of curcumin). At different time points (24, 48 and 72 h) each well was supplemented with 10% (*v*/*v*) MTT solution and incubated for 4 h. Formazan crystals were dissolved in acidified isopropanol (isopropanol, 0.1 N HCl, 0.1% Tween-20). The optical density was measured at 570 nm by using a microplate reader (Tecan Infinite F200PRO). The 50% inhibitory concentration (IC_50_) of curcumin-loaded PGS-NPs and free curcumin on HeLa cells was calculated at 72 h using Graph Pad Prism software. 

#### 3.8.2. RNA Extraction and Real-Time RT-PCR Analysis

Total RNA was isolated from cells (1 × 10^6^ cells per well in 6-well plate) treated with unloaded and curcumin-loaded PGS-NPs (0.005 and 0.01 mg/mL) using Direct-Zol^TM^ RNA MiniPrep kit (Zymo Research, Irvine, CA, USA). About 1 μg of RNA was reverse-transcribed with the iScript cDNA Synthesis Kit (Bio-Rad, Hercules, CA, USA) and real-time RT-PCR was performed with the QuantiNova SYBR Green kit (Qiagen, Hilden, Germany) for gene expression in a CFX96 Real-time PCR detection system (Bio-Rad, Hercules, CA, USA). Transcript levels were normalized against the *GAPDH* gene and values were expressed as fold change over control sample treated with vehicle (CNT). Primer sequences are reported in Table 4. Data are presented as mean ± SEM.

#### 3.8.3. Western Blotting

HeLa cells (1 × 10^6^ cells per well in 6-well plate) treated with curcumin-loaded NPs (0.005 and 0.01 mg/mL of curcumin-loaded NPs) for 72 h were washed with PBS and then lysed in a buffer containing 50 mM Tris–HCl, pH 7.2, 0.1% sodium deoxycholate, 1% Triton X-100, 5 mM EDTA, 5 mM EGTA, 150 mM NaCl, 40 mM NaF, 2.175 mM NaVO_4_, 0.1% SDS, 0.1% aprotinin and 1 mM PMSF. Forty micrograms of protein underwent SDS–PAGE following transfer on PVDF membranes (Amersham™ Hybond^®^ P, Dasser Germany). Bands were detected using Immobilon ECL Western Blotting Substrate (ThermoFisher Scientific, Waltham, MA, USA) on a Chemidoc digital imaging machine (Bio-Rad). An anti-Cleaved Caspase-3 primary antibody (1:1000 in 1% BSA, Cell Signaling Technology, Inc. MA, USA) and an anti-PARP-1 primary antibody (1:1000 in 1% BSA, Santa Cruz Biotechnology, Dallas, TX, USA), were used both followed by a goat anti-rabbit horseradish peroxidase-conjugated secondary antibody (1:10,000; Abcam Cambridge CB2 0AX UK). Internal loading control was performed using the anti-**α**-Tubulin mouse primary antibody (1:10,000 in 1% BSA; Sigma-Aldrich) followed by a goat anti-mouse horseradish peroxidase-conjugated secondary antibody (1:10,000, Sigma-Aldrich). Densitometry quantification was performed with ImageJ Software (NIH) and expressed as ratio of cleaved caspase-3 and PARP to α-tubulin.

#### 3.8.4. Statistics

The statistical significance of differences in cell assays, gene and protein expression analyses were determined by one-way ANOVA followed by Tukey’s post hoc tests for multiple comparisons. The statistical analysis was carried out using the software GraphPad Prism 6 (GraphPad, San Diego, CA, USA). A value of *p* < 0.05 was considered significant.

## 4. Conclusions

Poly(glycerol sebacate) was, for the first time, exploited for the encapsulation of curcumin, with the aim to design a nanosized anticancer system characterized by high stability even under physiological conditions and strong biological activity through an easy, scalable and reproducible technique. Different experimental conditions have been tested, aimed to define the most promising formulations in terms of particle stability and size. Indeed, the curcumin-loaded sample PGS_5.0_C_0.5_ and the respective unloaded formulation PGS_5.0_Blank, were selected in virtue of their stability even when diluted in simulated cells medium. Stability and morphological analysis were performed through DLS and TEM analysis which allowed confirmation of their nanosized dimension of about 150 nm and spherical shape. Finally, curcumin-loaded PGS-NPs displayed a higher cytotoxic effect compared to free curcumin, thanks to the higher solubility in physiological conditions. Consistent with this, the developed curcumin-loaded PGS-NPs were capable of inducing apoptosis by promoting the expression of *p53*, its target gene, *p21*, involved in cell cycle arrest and the pro-apoptotic *Bax* gene. Finally, a reduction of *HPV E6* expression was observed, suggesting that curcumin-loaded PGS-NPs can exert a significant anti-HPV activity, thus possibly preventing pre-cancer lesions. Overall, our results show that curcumin-loaded PGS-NPs may represent a possible adjuvant therapy for delaying/treating cervical cancer cells. 

## Figures and Tables

**Table 1:** Effect of polymer concentration on PGS-NPs particle diameter at t = 0, after 7 days and after 14 days from sample preparation.

Run	Mean Diameter (nm)		
	0 Days	7 Days	14 Days
*PGS_0.1_Blank*	89 ± 1 PdI: 0.21	92 ± 2 PdI: 0.21	96 ± 2 PdI: 0.04
*PGS_0.1_C_0.01_*	81 ± 1 PdI: 0.25	225 ± 14 PdI: 0.73 (presence of macroaggregates)	-
*PGS_0.5_Blank*	123 ± 1 PdI: 0.01	120 ± 3 PdI: 0.01	119 ± 3 PdI: 0.08
*PGS_0.5_C_0.05_*	106 ± 2 PdI:0.21	136 ± 4 PdI:0.82 (presence of macroaggregates)	-
*PGS_1.0_Blank*	164 ± 4 PdI: 0.01	165 ± 3 PdI: 0.01	163 ± 4 PdI: 0.03
*PGS_1.0_C_0.1_*	158 ± 4 PdI:0.03	159 ± 6 PdI:0.03	130 ± 4 PdI: 0.05
*PGS_5.0_Blank*	234 ± 6 PdI: 0.02	229 ± 6 PdI: 0.03	230 ± 6 PdI: 0.01
*PGS_5.0_C_0.5_*	211 ± 2 PdI: 0.001	173 ± 4 PdI: 0.02	169 ± 2 PdI: 0.02

PdI: Polydispersity index.

**Table 2:** PGS_1.0_C_0.1_, PGS_5.0_C_0.5_ recorded mean diameters at different times from sample preparation in deionized water and DMEM.

**Run in Deionized Water**	**Mean Diameter (nm)**			
	**Day 0**	**Day 1**	**Day 2**	**Day 3**
PGS_1.0_C_0.1_ *[0.2 mg/mL]*	108 ± 3 PdI: 0.23	87 ± 1 PdI: 0.05	85 ± 2 PdI: 0.06	84 ± 2 PdI: 0.06
PGS_5.0_C_0.5_ *[0.2 mg/mL]*	136 ± 2 PdI: 0.07	136 ± 2 PdI: 0.07	136 ± 1 PdI: 0.02	135 ± 1 PdI: 0.02
**Run in DMEM**	**Mean Diameter (nm)**			
	**Day 0**	**Day 1**	**Day 2**	**Day 3**
PGS_1.0_C_0.1_ *[0.2 mg/mL]*	148 ± 2 PdI: 0.20	137 ± 2 PdI: 0.21	129 ± 1 PdI: 0.22	127 ± 1 PdI: 0.24
PGS_5.0_C_0.5_ *[0.2 mg/mL]*	137 ± 1 PdI: 0.17	129 ± 1 PdI: 0.19	129 ± 1 PdI: 0. 21	129 ± 1 PdI: 0.19

PdI: Polydispersity index.

**Table 3:** Tested formulation parameters for the preparation of PGS-based NPs by nanoprecipitation.

	Organic Phase Composition	
Sample	PGS	Curcumin
*PGS_0.1_Blank*	1 mg [0.1 mg/mL]	\
*PGS_0.1_C_0.01_*	1 mg [0.1 mg/mL]	0.1 mg [0.01 mg/mL]
*PGS_0.5_Blank*	5 mg [0.5 mg/mL]	\
*PGS_0.5_C_0.05_*	5 mg [0.5 mg/mL]	0.5 mg [0.05 mg/mL]
*PGS_1.0_Blank*	10 mg [1 mg/mL]	\
*PGS_1.0_C_0.1_*	10 mg [1 mg/mL]	1 mg [0.1 mg/mL]
*PGS_5.0_Blank*	50 mg [5 mg/mL]	\
*PGS_5.0_C_0.5_*	50 mg [5 mg/mL]	5 mg [0.5 mg/mL]

**Table 4:** Primer sequences used for real-time RT-PCR.

Primer	Primer Sequence 5′–3′
hs-HPV18-E6 fw	GTGCCAGAAACCGTTGAATCC
hs-HPV18-E6 rv	AGTCTTTCCTGTCGTGCTCG
hs-p53 fw	GAGGGATGTTTGGGAGATGTAA
hs-p53 rv	CCCTGGTTAGTACGGTGAAGTG
hs-p21 fw	GTCACTGTCTTGTACCCTTGTG
hs-p21 rv	AGAAATCTGTCATGCTGGTCTGC
hs-Bax fw	CATGGGCTGGACATTGGACTT
hs-Bax rv	AGGGACATCAGTCGCTTCAGT
hs-Gapdh fw	GCCTCAAGATCATCAGCAATGC
hs-Gapdh rv	CCACGATACCAAAGTTGTCATGG

fw, forward; rv, reverse.

## Data Availability

Not applicable.

## Supplementary Materials

The following supporting information can be downloaded at: https://www.mdpi.com/article/10.3390/molecules27206997/s1.

## Sample Availability

Samples of the compounds are not available from the authors.

## References

[1] Cancer: Data and Statistics. https://www.euro.who.int/en/health-topics/noncommunicable-diseases/cancer/data-and-statistics.

[2] Cai S., Yang Q., Bagby T.R., Forrest M.L. (2011). Lymphatic drug delivery using engineered liposomes and solid lipid nanoparticles. Adv. Drug Deliv. Rev..

[3] Zhao K., Li D., Shi C., Ma X., Rong G., Kang H., Wang X., Sun B. (2016). Biodegradable Polymeric Nanoparticles as the Delivery Carrier for Drug. Curr. Drug Deliv..

[4] Masserini M. (2013). Nanoparticles for Brain Drug Delivery. ISRN Biochem..

[5] Owens D.E., Peppas N.A. (2006). Opsonization, biodistribution, and pharmacokinetics of polymeric nanoparticles. Int. J. Pharm..

[6] Stylianopoulos T. (2013). EPR-effect: Utilizing size-dependent nanoparticle delivery to solid tumors. Ther. Deliv..

[7] Cho K., Wang X., Nie S., Chen Z., Shin D.M. (2008). Therapeutic nanoparticles for drug delivery in cancer. Clin. Cancer Res..

[8] Maeda H., Nakamura H., Fang J. (2013). The EPR effect for macromolecular drug delivery to solid tumors: Improvement of tumor uptake, lowering of systemic toxicity, and distinct tumor imaging in vivo. Adv. Drug Deliv. Rev..

[9] Chiellini E., Covolan V.L., Orsini L.M., Solaro R. (2003). Polymeric nanoparticles based on polylactide and related copolymers. Macromolecular Symposia.

[10] Vogt L., Ruther F., Salehi S., Boccaccini A.R. (2021). Poly(Glycerol Sebacate) in Biomedical Applications—A Review of the Recent Literature. Adv. Healthc. Mater..

[11] Wu Z., Jin K., Wang L., Fan Y. (2021). A Review: Optimization for Poly(glycerol sebacate) and Fabrication Techniques for Its Centered Scaffolds. Macromol. Biosci..

[12] Loh X.J., Abdul Karim A., Owh C. (2015). Poly(glycerol sebacate) biomaterial: Synthesis and biomedical applications. J. Mater. Chem. B.

[13] Sha D., Wu Z., Zhang J., Ma Y., Yang Z., Yuan Y. (2021). Development of modified and multifunctional poly(glycerol sebacate) (PGS)-based biomaterials for biomedical applications. Eur. Polym. J..

[14] Louage B., Tack L., Wang Y., De Geest B.G. (2017). Poly(glycerol sebacate) nanoparticles for encapsulation of hydrophobic anti-cancer drugs. Polym. Chem..

[15] Sivanesan D., Verma R.S., Prasad E. (2021). 5FU encapsulated polyglycerol sebacate nanoparticles as anti-cancer drug carriers. RSC Adv..

[16] Hawley A.E., Davis S.S., Illum L. (1995). Targeting of colloids to lymph nodes: Influence of lymphatic physiology and colloidal characteristics. Adv. Drug Deliv. Rev..

[17] Tamvakopoulos C., Dimas K., Sofianos Z.D., Hatziantoniou S., Han Z., Liu Z.L., Wyche J.H., Pantazis P. (2007). Metabolism and anticancer activity of the curcumin analogue, dimethoxycurcumin. Clin. Cancer Res..

[18] Subramani P.A., Panati K., Narala V.R. (2017). Curcumin Nanotechnologies and Its Anticancer Activity. Nutr. Cancer.

[19] Allegra A., Innao V., Russo S., Gerace D., Alonci A., Musolino C. (2017). Anticancer Activity of Curcumin and Its Analogues: Preclinical and Clinical Studies. Cancer Investig..

[20] Aggarwal B.B., Kumar A., Bharti A.C. (2003). Anticancer potential of curcumin: Preclinical and clinical studies. Anticancer Res..

[21] Basniwal R.K., Khosla R., Jain N. (2014). Improving the anticancer activity of curcumin using nanocurcumin dispersion in water. Nutr. Cancer.

[22] Yallapu M.M., Khan S., Maher D.M., Ebeling M.C., Sundram V., Chauhan N., Ganju A., Balakrishna S., Gupta B.K., Zafar N. (2014). Anti-cancer activity of curcumin loaded nanoparticles in prostate cancer. Biomaterials.

[23] D’Angelo N.A., Noronha M.A., Kurnik I.S., Câmara M.C.C., Vieira J.M., Abrunhosa L., Martins J.T., Alves T.F.R., Tundisi L.L., Ataide J.A. (2021). Curcumin encapsulation in nanostructures for cancer therapy: A 10-year overview. Int. J. Pharm..

[24] Hornig S., Heinze T., Becer C.R., Schubert U.S. (2009). Synthetic polymeric nanoparticles by nanoprecipitation. J. Mater. Chem..

[25] Errico C., Goñi-De-Cerio F., Alderighi M., Ferri M., Suarez-Merino B., Soroka Y., Frǔié-Zlotkin M., Chiellini F. (2012). Retinyl palmitate-loaded poly(lactide-co-glycolide) nanoparticles for the topical treatment of skin diseases. J. Bioact. Compat. Polym..

[26] Sung H., Ferlay J., Siegel R.L., Laversanne M., Soerjomataram I., Jemal A., Bray F. (2021). Global Cancer Statistics 2020: Globocan Estimates of Incidence and Mortality Worldwide for 36 Cancers in 185 Countries. CA Cancer J. Clin..

[27] Burmeister C.A., Khan S.F., Schäfer G., Mbatani N., Adams T., Moodley J., Prince S. (2022). Cervical cancer therapies: Current challenges and future perspectives. Tumour Virus Res..

[28] Maleki Dana P., Sadoughi F., Asemi Z., Yousefi B. (2022). The role of polyphenols in overcoming cancer drug resistance: A comprehensive review. Cell. Mol. Biol. Lett..

[29] Wang Y., Ameer G.A., Sheppard B.J., Langer R. (2002). A tough biodegradable elastomer. Nat. Biotechnol..

[30] Chen Q.Z., Bismarck A., Hansen U., Junaid S., Tran M.Q., Harding S.E., Ali N.N., Boccaccini A.R. (2008). Characterisation of a soft elastomer poly(glycerol sebacate) designed to match the mechanical properties of myocardial tissue. Biomaterials.

[31] Rai R., Tallawi M., Grigore A., Boccaccini A.R. (2012). Synthesis, properties and biomedical applications of poly(glycerol sebacate) (PGS): A review. Prog. Polym. Sci..

[32] Rafiee Z., Nejatian M., Daeihamed M., Jafari S.M. (2019). Application of different nanocarriers for encapsulation of curcumin. Crit. Rev. Food Sci. Nutr..

[33] Mukerjee A., Vishwanatha J.K. (2009). Formulation, characterization and evaluation of curcumin-loaded PLGA nanospheres for cancer therapy. Anticancer Res..

[34] Sari T.P., Mann B., Kumar R., Singh R.R.B., Sharma R., Bhardwaj M., Athira S. (2015). Preparation and characterization of nanoemulsion encapsulating curcumin. Food Hydrocoll..

[35] De Moraes Carvalho D., Takeuchi K.P., Geraldine R.M., de Moura C.J., Torres M.C.L. (2015). Production, solubility and antioxidant activity of curcumin nanosuspension. Food Sci. Technol..

[36] Deng L., Kang X., Liu Y., Feng F., Zhang H. (2017). Effects of surfactants on the formation of gelatin nanofibres for controlled release of curcumin. Food Chem..

[37] Petroni K., Trinei M., Fornari M., Calvenzani V., Marinelli A., Micheli L.A., Pilu R., Matros A., Mock H.P., Tonelli C. (2017). Dietary cyanidin 3-glucoside from purple corn ameliorates doxorubicin-induced cardiotoxicity in mice. Nutr. Metab. Cardiovasc. Dis..

