# Supplementary material for: Synthesis and Characterization of Curcumin-Loaded Nanoparticles of Poly(Glycerol Sebacate): A Novel Highly Stable Anticancer System
Source: Molecules. 2022 Oct 18;27(20):6997. doi: 10.3390/molecules27206997 (PMC9606863; doi:10.3390/molecules27206997)
Supplement: Supplementary file 1 [file molecules-27-06997-s001.zip › molecules-1938533-supplementary.pdf]

Supporting Information

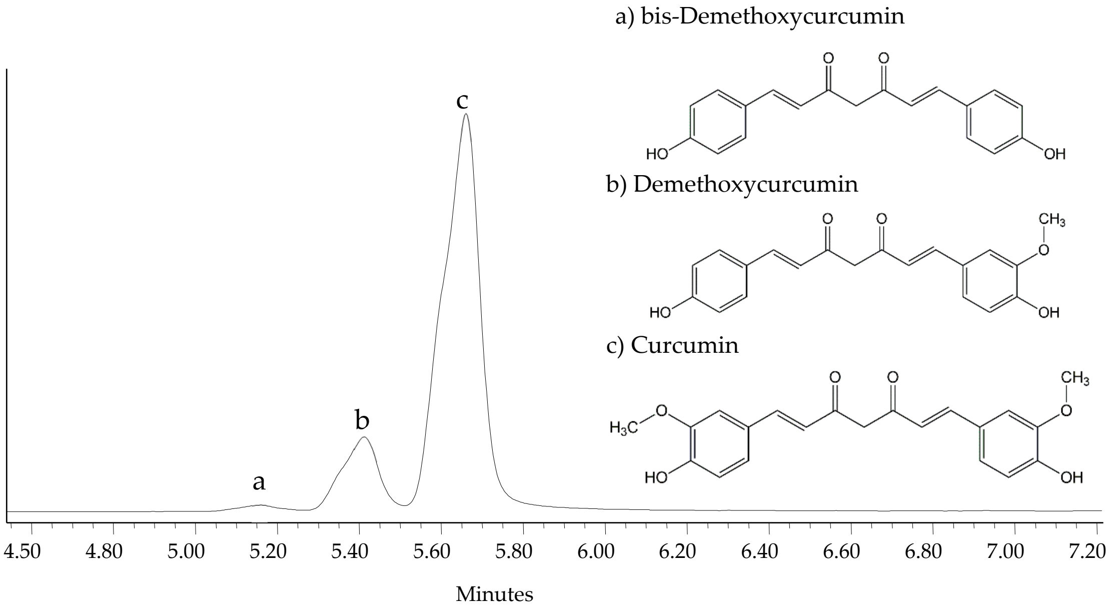

Figure S1. UPLC chromatogram of curcumin extract recorded at 445 nm. Inset: a) bis-Demethoxycurcumin, b) Demethoxycurcumin, c) Curcumin chemical structures.

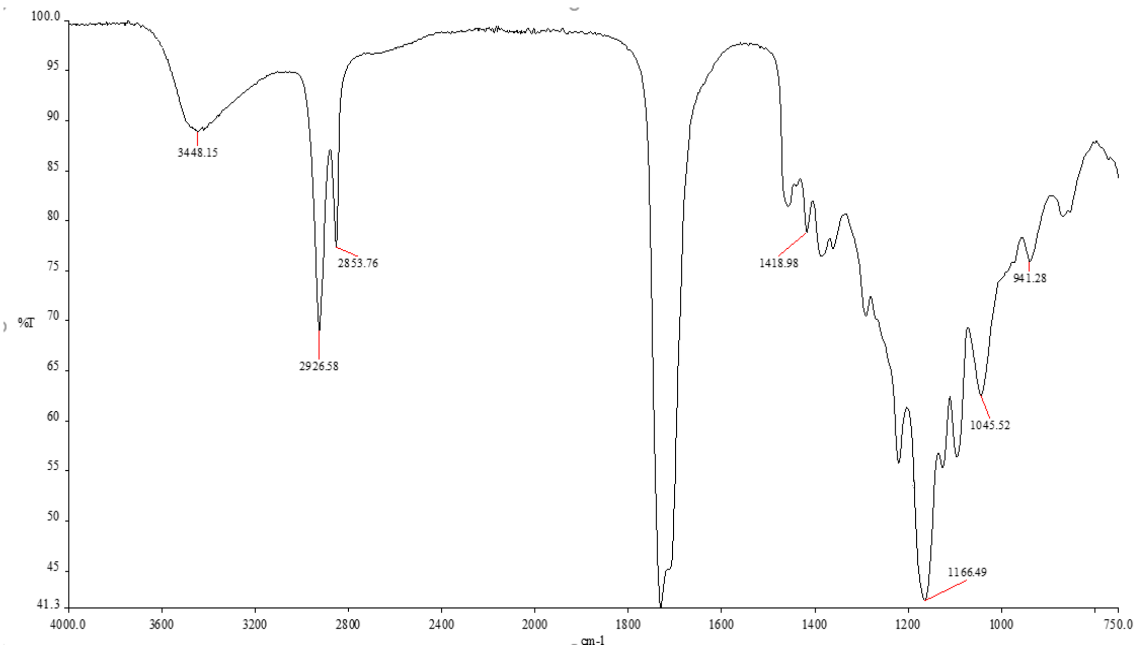

Figure S2. FT-IR spectrum of PGS

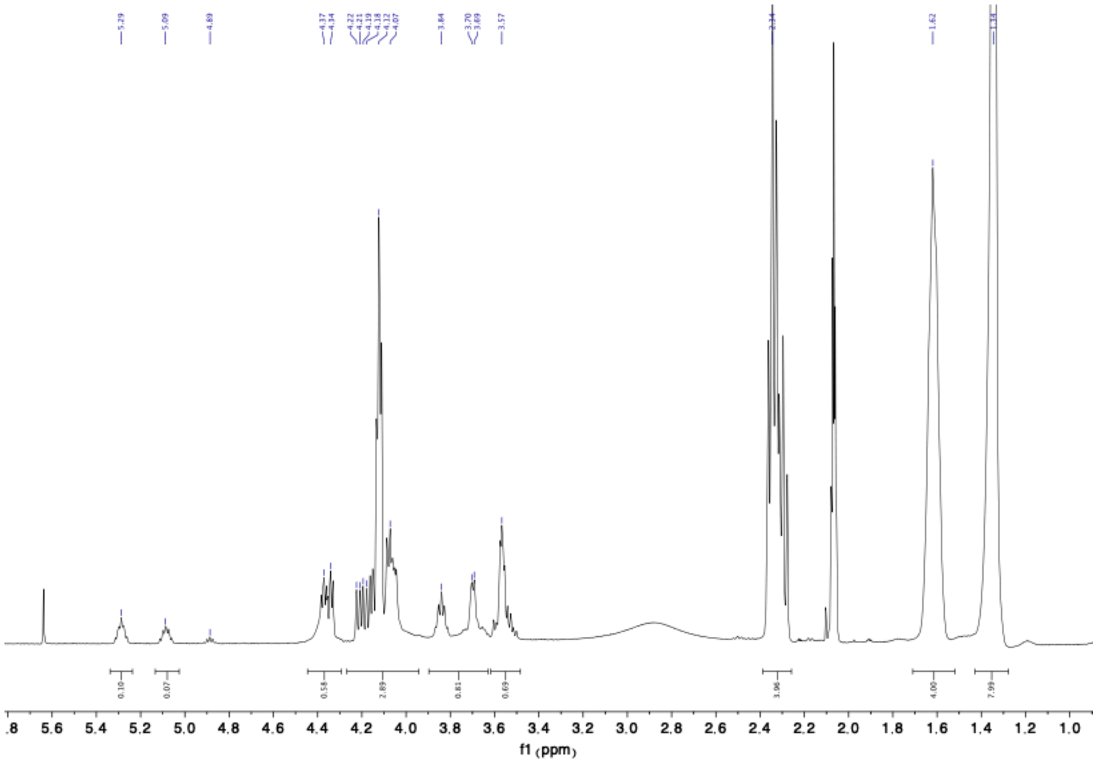

Figure S3. ^{1}H NMR spectrum of PGS

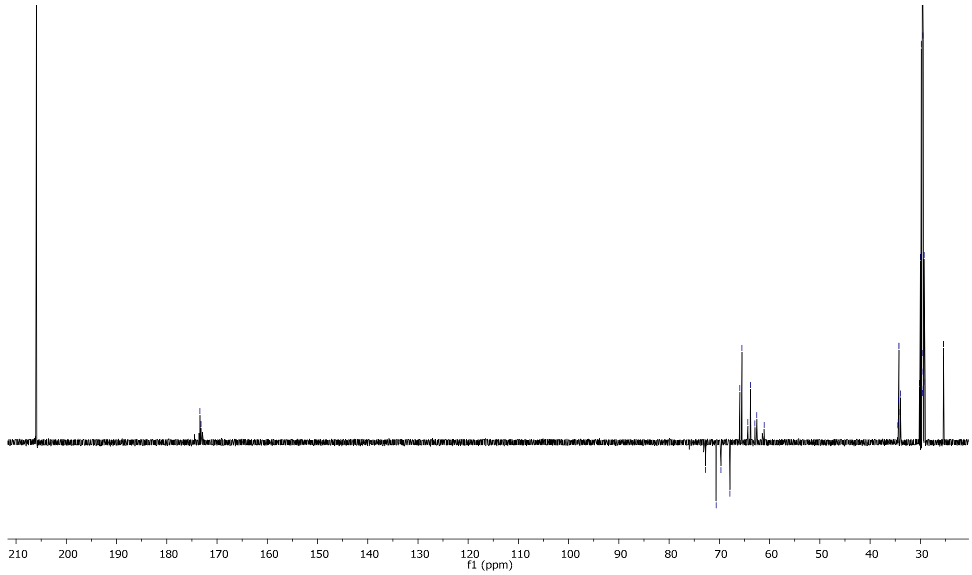

Figure S4. ^{13}C APT NMR spectrum of PGS

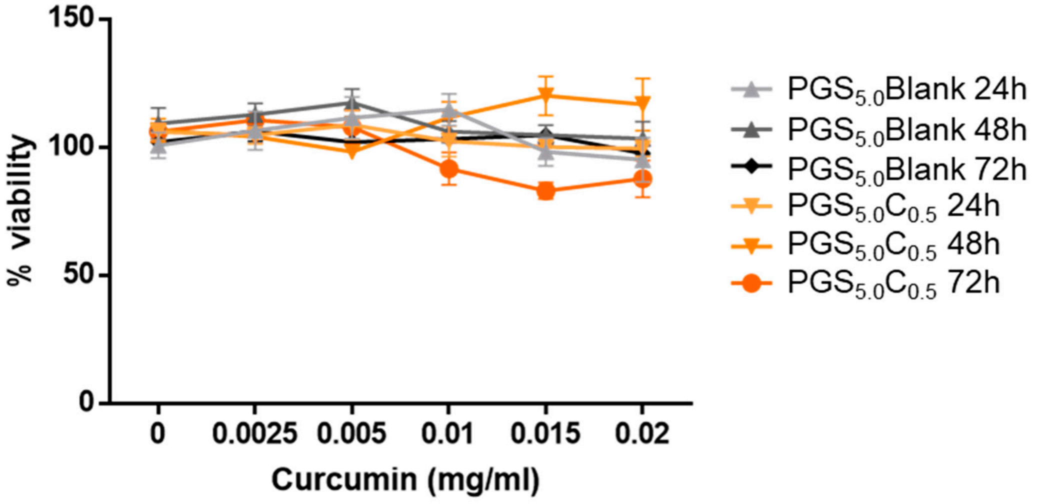

Figure S5. Cytotoxicity assay in NIH-3T3 cells with different concentrations of $PGS_{5.0}$Blank and $PGS_{5.0}C_{0.5}$ dissolved in sterile dH_2O
